# Functional Crosstalk between Type I and II Interferon through the Regulated Expression of STAT1

**DOI:** 10.1371/journal.pbio.1000361

**Published:** 2010-04-27

**Authors:** Daniel J. Gough, Nicole L. Messina, Linda Hii, Jodee A. Gould, Kanaga Sabapathy, Ashley P. S. Robertson, Joseph A. Trapani, David E. Levy, Paul J. Hertzog, Christopher J. P. Clarke, Ricky W. Johnstone

**Affiliations:** 1 Peter MacCallum Cancer Centre, East Melbourne, Victoria, Australia; 2 University of Melbourne, Melbourne, Victoria, Australia; 3 New York University Langone Medical Center, New York, New York, United States of America; 4 Centre for Innate Immunity and Infectious Disease, Monash Institute of Medical Research, Monash University, Melbourne, Victoria, Australia; 5 National Cancer Centre, Singapore, Singapore; Washington University School of Medicine, United States of America

## Abstract

Autocrine priming of cells by small quantities of constitutively produced type I interferon (IFN) is a well-known phenomenon. In the absence of type I IFN priming, cells display attenuated responses to other cytokines, such as anti-viral protection in response to IFNγ. This phenomenon was proposed to be because IFNα/β receptor1 (IFNAR1) is a component of the IFNγ receptor (IFNGR), but our new data are more consistent with a previously proposed model indicating that regulated expression of STAT1 may also play a critical role in the priming process. Initially, we noticed that DNA binding activity of STAT1 was attenuated in *c-Jun* ^−*/*−^ fibroblasts because they expressed lower levels of STAT1 than *wild-type* cells. However, expression of STAT1 was rescued by culturing *c-Jun* ^−*/*−^ fibroblasts in media conditioned by *wild-type* fibroblasts suggesting they secreted a STAT1-inducing factor. The STAT1-inducing factor in fibroblast-conditioned media was IFNβ, as it was inhibited by antibodies to IFNAR1, or when IFNβ expression was knocked down in *wild-type* cells. IFNAR1^−/−^ fibroblasts, which cannot respond to this priming, also expressed reduced levels of STAT1, which correlated with their poor responses to IFNγ. The lack of priming in IFNAR1^−/−^ fibroblasts was compensated by over-expression of STAT1, which rescued molecular responses to IFNγ and restored the ability of IFNγ to induce protective anti-viral immunity. This study provides a comprehensive description of the molecular events involved in priming by type I IFN. Adding to the previous working model that proposed an interaction between type I and II IFN receptors, our work and that of others demonstrates that type I IFN primes IFNγ-mediated immune responses by regulating expression of STAT1. This may also explain how type I IFN can additionally prime cells to respond to a range of other cytokines that use STAT1 (e.g., IL-6, M-CSF, IL-10) and suggests a potential mechanism for the changing levels of STAT1 expression observed during viral infection.

## Abstract 2

Small "priming" quantities of type I interferon enhance cellular responses to type II interferon by maintaining basal levels of STAT1, explaining the observed crosstalk between these two cytokines.

## Author Summary

Cells of the immune system release interferons (IFNs) in response to pathogens or tumor cells; these proteins signal to other immune cells to initiate the body's defense mechanisms. The two classes of IFNs—types I and II—have different receptors and distinct effects on the cells; however, there is “crosstalk” between them. In particular, small quantities of type I IFN can “prime” cells to produce a robust response to type II IFN. In this paper, we provide evidence to explain the molecular basis of this crosstalk. We show that continuous expression of the transcriptional activator c-Jun is responsible for producing basal, priming levels of a type I IFN; this signals to immune cells with the type I IFN receptor (IFNAR1) to maintain expression of STAT1 inside these cells. STAT1 is a key factor for immune cell responses to type II IFN. Thus, signaling by low levels of type I IFN primes the cells with sufficient STAT1 to respond robustly to a subsequent type II IFN signal. This work provides an alternative explanation of the priming phenomenon to a previous proposal that the ligand-bound type I receptor, IFNAR1, acts as a component of the type II IFN receptor.

## Introduction

Although type I and type II interferons (IFNs) have distinct roles in immune responses, there is substantial overlap between the genes and cellular responses they regulate. It has been known for some time that many cells secrete small priming quantities of type I IFNs that facilitate more potent responses to subsequent stimuli [1]–[3]. Moreover, cellular responses to CSF-1 or IFNγ can be affected by neutralizing type I IFN antibodies or knockout of type I IFN-Receptors (IFNAR) [2],[4],[5]. Notably, the protective anti-viral effects of IFNγ were much less potent in *IFNAR1* ^−*/*−^ than *wild-type* fibroblasts which appeared to be caused by a lack of type I IFN priming [4],[5]. The molecular events that underpin these priming events have not been fully characterized, although it has been proposed that type I and II IFNs shared receptor components [5]. However, as the majority of responses to type I and II IFNs require the expression of the STAT1 transcription factor [6], this is also a possible point of crosstalk between them.

STAT1 is a key mediator of cytokine-induced gene expression as it is activated either as homo- or heterodimer with other STATs by many cytokines including type I and type II IFNs, interleukin (IL)-6 and IL-10. STAT1 activity is of particular importance to the IFN system as STAT1^−/−^ mice display many similar phenotypes to mice lacking IFNAR1 or the IFN Receptor (IFNGR)1. In particular, anti-viral, anti-mycobacterial, and anti-tumor responses are compromised [6]–[9]. Induction of STAT1 expression is a potential explanation for the priming activity of type I IFN because it is an IFN-stimulated gene (ISG) itself [10]–[12] and its 5′ promoter region contains an IRF/gamma activated sequence (GAS) element bound by IFN-stimulated transcription factors [13]. Inducing the expression of STAT1 would increase the pool of this factor available for activation by IFNγ. Consistent with such a hypothesis, low expression of STAT1 correlated with IFN-resistance in melanoma samples when compared to surrounding normal tissue [14].

In unstimulated cells, STAT1 resides in the cytoplasm as a latent factor that is activated by a series of post-translational modifications initiated when it is recruited to cytokine receptors following receptor ligation [15]. At the receptor, STAT1 is phosphorylated on tyrosine 701, by Janus family kinase (JAK)s, which facilitates its dimerization either with other STAT1 molecules or other STAT proteins depending on the cytokine receptor. In addition, STAT1 proteins are phosphorylated on serine 727 prior to nuclear translocation which is essential for their full transcriptional activity [16]. Conversely, STAT1 activity is negatively regulated by phosphatases, SOCS proteins, and the SUMO ligase Protein Inhibitor of Activated STAT (PIAS)1 [15].

Recently, in the course of our studies on IFNγ-activated AP-1 DNA binding, we noticed that IFNγ-induced GAS DNA binding was suppressed in *c-Jun* ^−*/*−^ cells compared to *wild-type* cells [17] and this correlated reduced levels of STAT1 in *c-Jun* ^−*/*−^ cells. The level of STAT1 expression in *c-Jun* ^−*/*−^ murine embryonic fibroblasts (MEFs) were restored to *wild-type* levels following culture in media conditioned by *wild-type* fibroblasts suggesting that *c-Jun* deficiency caused the disruption of an autocrine/paracrine loop that regulated STAT1 expression. The STAT1-inducing component of media conditioned by *wild-type* fibroblasts was IFNβ, because the activity could be blocked by neutralizing antibodies directed against type I IFN and antibodies used were raised against IFNAR and attenuated by targeted knockdown of IFNβ by RNA interference (RNAi). While c-Jun has been demonstrated to co-operate with ATF-2, IRF-3, and NFκB for virus-induced production of IFNβ [18], to our knowledge our studies are the first to demonstrate that c-Jun is necessary for basal expression of low-level IFNβ. Fibroblasts in which this autocrine/paracrine loop was disrupted by the loss of components of type I IFN receptors also express lower levels of STAT1. As many biological functions of IFN require STAT1 [6],[7], this suggested that previous observations of attenuated responses to IFN in IFNAR1^−/−^ cells may be related to the reduced STAT1 expression that has been observed [19]. Consistent with this hypothesis, restoring STAT1 expression in *IFNAR1* ^−*/*−^ fibroblasts rescued IFNγ-induced gene transcription and anti-viral properties.

In summary, this study provides evidence of an autocrine/paracrine stimulatory loop that requires the expression of c-Jun, IFNβ, and IFNAR to regulate the expression of STAT1. Importantly, this basal IFNβ production occurs via a mechanism distinct from the pathogen-stimulated IFNβ production mediated by IRF and NFκB pathways [18]. One model to explain crosstalk between type I and II IFNs states that type I and II IFN-R physically interact in a ligand-dependant manner, such that the presence of type I IFNs is essential for a fully competent IFNγ response [5]. Herein, we demonstrated that attenuated IFNγ-mediated gene induction and an associated defective anti-viral response to IFNγ that is observed in IFNAR1-deficient cells can be rescued by re-expressing STAT1 and is therefore independent of IFNAR1. We propose that an alternative model to explain the functional synergy between type I and II IFNs is based on the regulated expression of STAT1 via c-Jun-mediated production of basal levels of IFNβ.

## Results

### STAT1 Expression Is Attenuated in c-Jun^−/−^ MEFs

**Figure 1:**
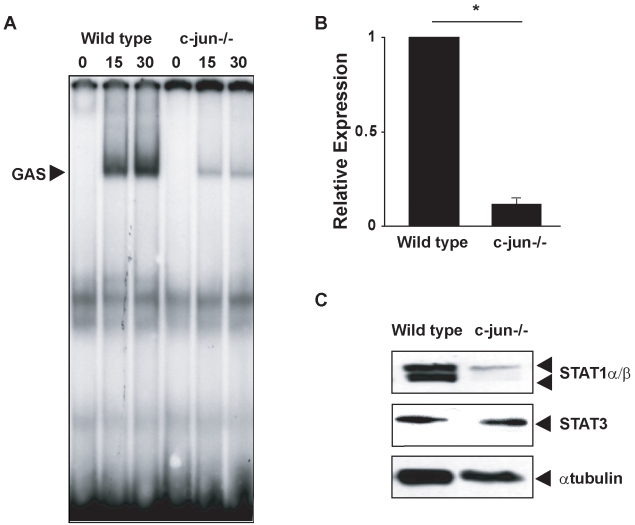
STAT1 DNA binding and expression is attenuated in c-Jun-deficient MEFs. (A) EMSAs were performed using radiolabeled oligonucleotides containing a GAS consensus sequence, and nuclear extracts from *wild-type* or *c-Jun* ^−*/*−^ MEFs treated with 100 IU/mL IFNγ for indicated times. (B) RNA was extracted from *wild-type* or *c-Jun* ^−*/*−^ MEFs, cDNA synthesized, and qRT-PCR performed with primers complementary to murine *STAT1*. Histograms represent mean and error bars the SEM of four independent experiments and are expressed relative to the levels detected in *wild-type* cells (* *p*<0.05). (C) SDS-PAGE and Western blotting with antibodies against STAT1 and STAT3 were performed using whole cell extracts from *wild-type* or *c-Jun* ^−*/*−^ MEFs. As a control, the expression of α-tubulin was also tested by Western blot.

In the course of our studies of IFN-induced signaling and gene expression, we performed elecrophoretic mobility shift assays (EMSAs) assessing GAS binding species in nuclear extracts from IFNγ-stimulated *wild-type* and *c-Jun* ^−*/*−^ MEFs. A GAS binding complex was detected in both *wild-type* and matched *c-Jun* ^−*/*−^ MEFs following 15–30 min of exposure to IFNγ, however in the absence of c-Jun, IFNγ-induced GAS binding activity was markedly attenuated (Figure 1A). The decrease in GAS binding activity in *c-Jun* ^−*/*−^ MEFs was a consequence of reduced expression of STAT1. Both *STAT1* mRNA and protein were ∼10-fold lower in *c-Jun* ^−*/*−^ MEFs compared to *wild-type* cells (Figure 1B and C). However, expression of STATs was not globally affected, as expression of STAT3, another GAS-binding transcription factor, remained unchanged (Figure 1C). Reduced STAT1 expression was not a clone-specific phenomenon as similar results were obtained using an independently derived matched pair of *wild-type* and *c-Jun* ^−*/*−^ MEFs (Figure S1).

### c-Jun Maintains Levels of STAT1 Expression by Stimulating Autocrine Production of a Soluble Factor

**Figure 2:**
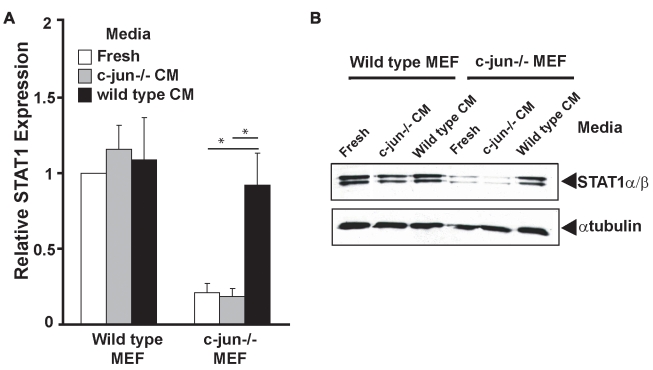
c-Jun is necessary for constitutive IFNβ secretion and subsequent maintenance of STAT1 expression. *Wild-type* or *c-Jun* ^−*/*−^ MEFs were cultured in fresh media (Fresh) or conditioned media from either c-Jun^−/−^ (c-Jun^−/−^ CM) or *wild-type* (wild-type CM) MEFs for 16 h. Cells were harvested, and (A) RNA was extracted and STAT1 mRNA expression assessed by qRT-PCR. Histograms represent mean and error bars the SEM of three independent experiments and are expressed relative to the levels detected in *wild-type* cells cultured in fresh media (* *p*<0.05 between indicated samples). (B) Whole cell lysates from treated cells were analyzed by SDS-PAGE and Western blot probed with an antibody specific for STAT1 and α-tubulin to confirm equivalent protein loading.

To determine if c-Jun could regulate STAT1 levels by inducing the secretion of a soluble factor that acted in autocrine/paracrine fashion to induce STAT1 expression, conditioned media from w*ild-type* or *c-Jun* ^−/−^ MEFs were cultured in (i) fresh media, (ii) media conditioned by *c-Jun* ^−*/*−^ MEFs, or (iii) media conditioned by *wild-type* MEFs. Cells were harvested after 16 h of culture in conditioned media and *STAT1* mRNA and protein expression was assessed. Expression of *STAT1* mRNA and protein was unaltered in *wild-type* MEFs cultured in fresh media or conditioned media from *wild-type* or *c-Jun* ^−*/*−^ MEFs (Figure 2A and B). In *c-Jun* ^−*/*−^ MEFs, basal expression of STAT1 was much lower than in *wild-type* cells and was not increased when the cells were cultured in either fresh media or conditioned media from *c-Jun* ^−*/*−^ MEFs (Figure 2A and B). In contrast, when *c-Jun* ^−*/*−^ MEFs were cultured in media conditioned by *wild-type* MEFs, *STAT1* mRNA and protein expression was induced almost to the levels observed in *wild-type* cells (Figure 2A and B). These data confirmed that fibroblasts secrete a c-Jun-dependent soluble factor that induces STAT1 expression through an autocrine/paracrine feedback loop.

### Constitutive Secretion of IFNβ Maintains Basal Expression of STAT1

**Figure 3:**
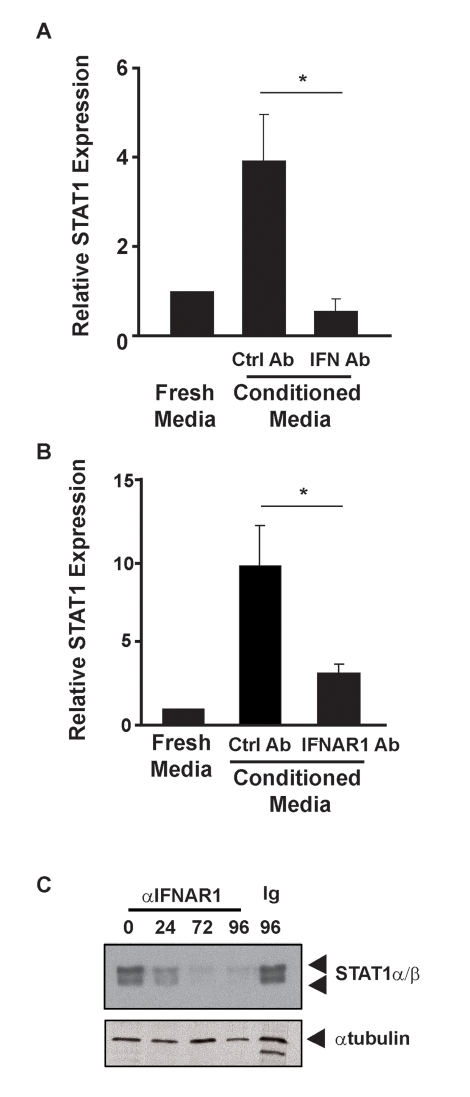
Antibodies that block type I IFN function modulate STAT1 expression. *c-Jun* ^−*/*−^ MEFs were cultured in fresh media or media conditioned by *wild-type* MEFs for 16 h. RNA was extracted and STAT1 mRNA expression assessed by qRT-PCR. (A) Conditioned media were supplemented with control antibody (Ctrl Ab) or type I IFN neutralizing antibody (IFN Ab). Histograms represent the mean and error bars the SEM of four independent experiments. (B) Conditioned media were supplemented with control antibody (Ctrl) or blocking antibody to IFNAR1 (IFNAR1 Ab). Histograms represent the mean and error bars the SEM of five independent experiments. In each case STAT1 levels are expressed relative to the levels detected in *c-Jun* ^−*/*−^ cells cultured in fresh media. (* *p*<0.05 between indicated samples). (C) *Wild-type* MEFs were cultured for various times in media containing antibodies to IFNAR1 or a control antibody for 96 h. At each time point, cells were harvested, whole cell extracts prepared, and STAT1 expression assessed by Western blot. Tubulin expression was assessed as a loading control.

Type I IFN is constitutively secreted from unstimulated fibroblasts and can induce STAT1 expression [10]. To determine if type I IFN was the STAT1-inducing active component of fibroblast conditioned media, *c-Jun* ^−*/*−^ MEFs were cultured in either fresh or conditioned media from *wild-type* cells in the presence of a type I IFN blocking antibody [20]. *STAT1* expression was increased in *c-Jun* ^−*/*−^ MEFs cultured in conditioned media from *wild-type* cells in the presence of control antibodies (Figure 3A) and this enhanced expression was entirely blocked by the presence of type I IFN neutralizing antibodies used at concentrations capable of neutralizing ∼5 IU/mL IFNβ. Additional studies (Figure 3B and C) revealed that the STAT1-inducing activity of *wild-type*-conditioned media was almost ablated by a blocking mAb raised against IFNAR1 [21]. Together, these data demonstrate that type I IFN is a component of conditioned media from *wild-type* cells that is necessary for the rescue of STAT1 expression in *c-Jun* ^−/−^ cells.

**Figure 4:**
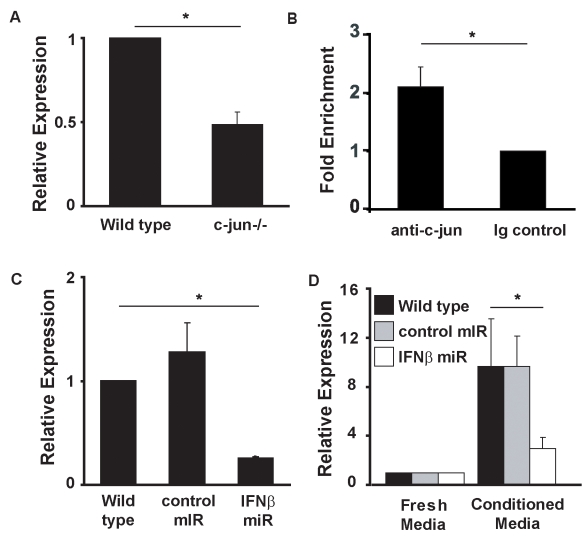
c-Jun binds to the *IFNβ* promoter and is required to maintain its expression in unstimulated cells. (A) RNA from *wild-type* and *c-Jun* ^−*/*−^ MEFs were assayed for IFNβ expression by qRT-PCR. Histograms represent mean and error bars the SEM of three independent experiments and are expressed relative to the levels detected in *wild-type* cells (arbitrarily set as one). (B) ChIP assays were performed using chromatin from *wild-type* MEFs using an anti c-Jun antibody or an isotype control antibody. Immuno-precipitated chromatin was assessed for enrichment of the *IFNβ* promoter by qRT-PCR using primers flanking PRD IV. Loading of c-Jun on PRD IV was calculated relative to isotype control. Histograms represent the mean and error bars the SEM of three independent experiments (* *p*<0.05 between indicated samples). (C) RNA was prepared from *wild-type* MEFs infected with recombinant lentivirus encoding microRNAs (miRs) targeting the IFNβ gene or a control miR. Expression of IFNβ was assessed by qRT-PCR. Histograms represent the mean and SEM of three independent experiments (* *p*<0.05 between indicated samples). (D) *c-Jun* ^−*/*−^ MEFs were treated with conditioned media from untransduced *wild-type* MEFs and MEFs transduced with lentivirus encoding either miRs targeting the IFNβ gene or a non-silencing miR or with fresh media. After 24 h, cells were harvested, RNA prepared, and STAT1 expression assessed by qRT-PCR. Histograms represent the mean and error bars the SEM of five independent experiments (* *p*<0.05 between indicated samples).

It has been reported that STAT1 levels are diminished in *IFNβ* ^−/−^ cells [22] indicating that IFNβ could be the key component of the conditioned media from *wild-type* cells shown to induce expression of STAT1 in *c-Jun* ^−/−^ MEFs. Treatment of *c-Jun* ^−/−^ MEFs with doses as low as 1 IU/mL IFNβ induced *STAT1* mRNA and doses between 5 and 10 IU/mL were sufficient to restore *STAT1* mRNA and protein expression to levels seen in *wild-type* cells (Figure S2A and B). *STAT1* mRNA levels were slightly increased in *wild-type* MEFs treated with IFNβ (Figure S2C), which is consistent with studies demonstrating that STAT1 expression is induced in fibosarcoma cell lines treated with IFNα or β [12] and in splenic leukocytes where STAT1 levels were increased following virus infection in a type I IFN-dependent manner [11]. Comparison of the levels of expression of IFNβ mRNA in *wild-type* and *c-Jun* ^−*/*−^ cells revealed that *c-Jun* ^−*/*−^ MEFs expressed ∼50% of the *wild-type* levels of *IFNβ* mRNA (Figure 4A). AP-1 sites are known to be important for inducible expression of IFN*β* [23], but little is known of what regulates constitutive production of type I IFN in unstimulated cultured fibroblasts. Chromatin immunoprecipitation (ChIP) assays on unstimulated *wild-type* and *c-Jun* ^−*/*−^ MEFs demonstrated a >2-fold increase in c-Jun bound to the murine *IFNβ* promoter when compared to Ig control samples (Figure 4B). Together, these data imply that expression of c-Jun and subsequent occupation of the *IFNβ* promoter by c-Jun is required for basal secretion of IFNβ.

To determine if IFNβ was the type I IFN necessary to maintain STAT1 expression, we used RNAi to knock down *IFNβ* in *wild-type* MEFs (Figure 4C) and assessed the ability of conditioned media from these cells to induce the expression of STAT1 mRNA in *c-Jun* ^−*/*−^ MEFs. As expected, *STAT1* mRNA levels were greater when *c-Jun* ^−*/*−^ MEFs were cultured in conditioned media from *wild-type* MEFs or from MEFs expressing a control knockdown vector than if these cells were cultured in fresh media (Figure 4D). In contrast, the ability of conditioned media from *wild-type* cells with RNAi-mediated knockdown of *IFNβ* to induce STAT1 expression in *c-Jun* ^−*/*−^ MEFs was significantly reduced (Figure 4D). These data confirm that IFNβ is expressed by unstimulated *wild-type* fibroblasts and is necessary for the maintenance of STAT1 expression.

### IFNAR-Deficient Cells Express Reduced Levels of STAT1

**Figure 5:**
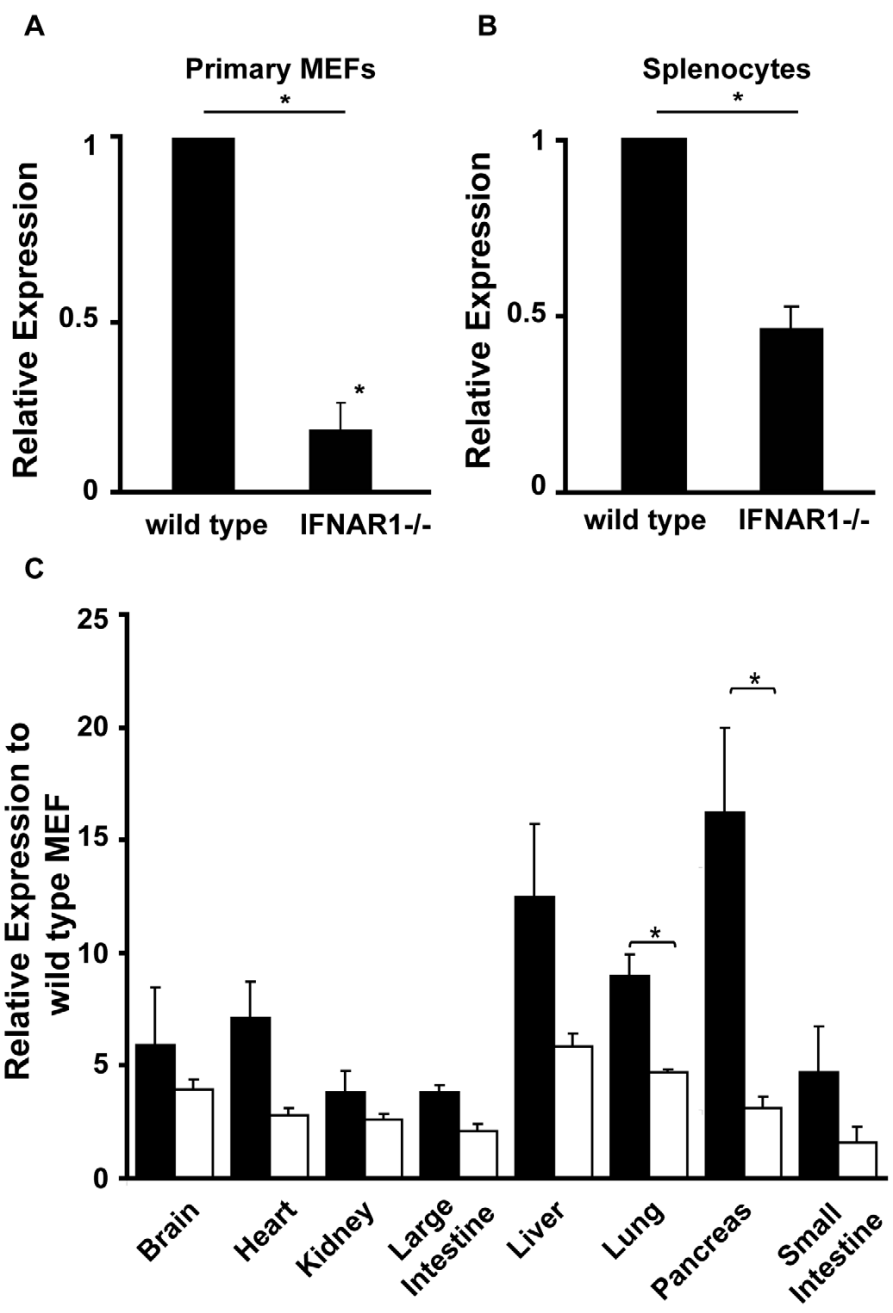
STAT expression in primary IFNAR-deficient cells. *Wild-type* and strain matched IFNAR1^−/−^ mice were sacrificed and RNA from (A) primary MEFs, (B) splenocytes, or (C) various indicated tissues was extracted, cDNA synthesized, and STAT1 mRNA expression assessed by qRT-PCR. STAT1 expression was normalized to untreated *wild-type* cells. Histograms show the mean of three independent experiments and error bars SEM (* *p*<0.05 between indicated samples).

As disruption of autocrine/paracrine stimulation by IFNβ affected the level of STAT1 expression in *c-Jun* ^−*/*−^ MEFs, we predicted that cells lacking either chain of the type I IFN receptor would also express less STAT1 than *wild-type* cells. Primary MEFs (Figure 5A) and splenocytes (Figure 5B) from either *IFNAR1* ^−*/*−^ or *IFNAR2* ^−*/*−^ (unpublished data) mice expressed significantly lower levels of *STAT1* than *wild-type* cells. We extended these studies to compare the expression of STAT1 across multiple tissues in *wild-type* versus *IFNAR1* ^−*/*−^ mice. As shown in Figure 5C, the levels of STAT1 were consistently reduced in all tissues from *IFNAR1* ^−*/*−^ mice compared to their wild-type counterparts, suggesting this defect may have broad physiological importance. Interestingly expression of STAT2 was also reduced in *IFNAR11* ^−*/*−^ MEFs while the levels of STAT3 were unaffected by knockout of the type I IFN receptor (Figure S3A).

Our model predicted that, unlike *c-Jun* deficiency that affected production of an autocrine stimulus, *IFNAR1* deficiency affects responses to the autocrine stimulus. In support of this model, *wild-type*-conditioned media was able to rescue the expression of STAT1 in *c-Jun* ^−*/*−^ MEFs, but in *IFNAR1* ^−*/*−^ MEFs *STAT1* expression was unaffected by culture in *wild-type*-conditioned media (Figure S3B). These data support the existence of an autocrine loop involving IFNβ that regulates basal STAT1 expression levels and suggest that defects in any part of this loop are likely to affect the expression of STAT1.

### Re-Expression of STAT1 in IFNAR-Deficient Cells Restores IFNγ Signaling and Gene Expression

**Figure 6:**
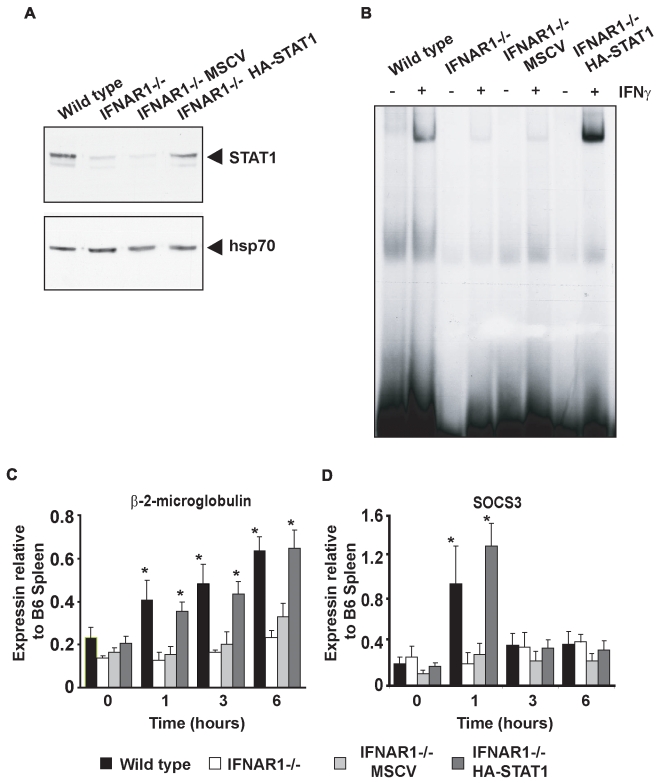
Restoration of STAT1α expression in IFNAR1^−/−^ MEFs rescues their IFNγ responsiveness. (A) Protein extracts from *wild-type* MEFs, IFNAR1^−/−^ MEFs, and IFNAR1^−/−^ MEFs transduced with empty vector (IFNAR1^−/−^ MSCV) or IFNAR1^−/−^ MEFs transduced with HA tagged STAT1α (IFNAR1^−/−^ STAT1) were subjected to SDS-PAGE and probed with an antibody specific to STAT1,and membranes were stripped and re-probed with antibodies specific to hsp70 as a loading control. (B) EMSAs were performed using radiolabeled oligonucleotides containing a GAS consensus sequence, and nuclear extracts from *wild-type* MEFs, IFNAR1^−/−^ MEFs, IFNAR1^−/−^ MEFs transduced with empty vector (IFNAR1^−/−^ MSCV), or IFNAR1^−/−^ MEFs transduced with HA tagged STAT1α (IFNAR1^−/−^ STAT1) treated in the presence or absence of 100 IU/mL IFNγ. (C, D) *Wild-type* MEFs, IFNAR1^−/−^ MEFs, and IFNAR1^−/−^ MEFs transduced with empty vector (IFNAR1^−/−^ MSCV) or IFNAR1^−/−^ MEFs transduced with HA-tagged STAT1α (IFNAR1^−/−^ STAT1) were treated with 100 IU/mL IFNγ for 0, 1, or 6 h. RNA was extracted, cDNA synthesized, and qRT-PCR performed with primers specific for *β-2-microglobulin* and *SOCS3*. mRNA levels are expressed relative to those of *wild-type* C57/BL6 (B6) splenocytes. Histograms represent the mean and error bars the standard error of four independent experiments. (* *p*<0.05 for samples that were significantly induced).

STAT1 is important for not only IFNα/β signaling but also the signaling of several other cytokines, including IFNγ [15]. The expression of approximately two-thirds of IFNγ-induced genes is dependent upon STAT1 expression, however not all IFNγ-mediated biological responses are entirely dependent on STAT1 expression [17],[24]. It has previously been reported that *IFNAR1* ^−*/*−^cells are refractory to IFNγ treatment due to the proposed interaction between IFNAR1 and IFNGR [5]. To determine if decreased expression of STAT1 may confer the observed decrease of IFNγ-mediated responses in IFNAR1^−*/*−^ cells, STAT1 levels were restored in these cells by retroviral transduction (Figure 6A). GAS binding activity was assessed by EMSA using nuclear extracts from IFNγ-treated *wild-type* MEFs, *IFNAR1* ^−*/*−^ MEFs, and *IFNAR1* ^−*/*−^ MEFs reconstituted with empty vector (*IFNAR1* ^−*/*−^ *MSCV*) or STAT1 (*IFNAR1* ^−*/*−^ *HA-STAT1*). Consistent with previous studies [5],[25], IFNγ induced less GAS binding in *IFNAR1* ^−*/*−^ cells than *wild-type* cells (Figure 6B). This low level of GAS binding was also observed in cells transduced with empty vector but was rescued in cells reconstituted with HA-STAT1α. These data demonstrated that the reduced GAS binding observed in *IFNAR1* ^−*/*−^ cells was caused by reduced STAT1 expression rather than being a direct consequence of IFNAR1 deficiency.

Previous studies demonstrated that IFNγ-induced gene expression was attenuated in *IFNAR1* ^−*/*−^ cells [5]. We therefore assessed the impact of re-expression of STAT1α in IFNAR1^−/−^ cells upon the IFNγ-induced expression of genes such as *β-2-microglobulin* and *SOCS3* that require STAT1 expression [26]. Both genes were induced in response to IFNγ in *wild-type* cells, although with differing kinetic profiles, but induction was weak or absent in *IFNAR1* ^−*/*−^ cells. IFNγ-induced expression of both *β-2-microglobulin* and *SOCS3* was restored in cells that re-expressed HA-STAT1α, but not in cells transduced with an empty vector (Figure 6C, D). Similar results were observed when other IFNγ-responsive genes were tested (Figure S4).

### Expression of STAT1 in IFNAR-Deficient Cells Restores Their Protective Anti-Viral Response Following Treatment with IFNγ

**Figure 7:**
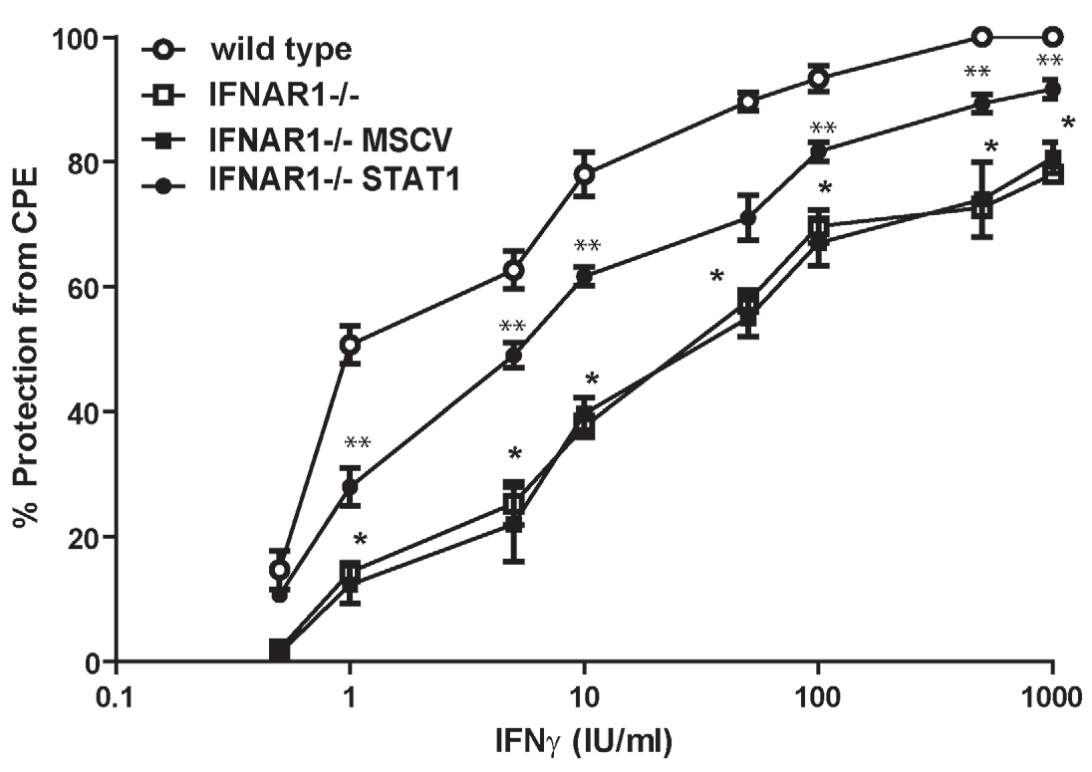
Restoration of STAT1α expression in IFNAR1^−/−^ MEFs rescues the ability of IFNγ to protect cells from cytopathic virus. *Wild-type* MEFs, *IFNAR1* ^−*/*−^ MEFs, and *IFNAR1* ^−*/*−^ MEFs transduced with vector (*IFNAR1* ^−*/*−^ *MSCV*) or *IFNAR1* ^−*/*−^ MEFs transduced with HA-tagged STAT1α (*IFNAR1* ^−*/*−^ *STAT1*) were infected with EMCV (0.1 moi) in the presence or absence of various doses of IFNγ. After 24 h, cells were stained with crystal violet and viability assessed by measuring OD_550_ of the solubilized stain. The effect of IFNγ was determined by comparison of OD_550_ with samples of known viability. Data points are the mean and error bars represent standard error from three independent experiments. (* *p*<0.05 between *wild-type* and *IFNAR1* ^−*/*−^ ** *p*<0.05 between *wild-type* and *IFNAR1* ^−*/*−^).

In order to determine whether the reduced levels of STAT1 in *IFNAR1* ^−*/*−^ cells could affect biological responses to IFNγ, we investigated whether re-expression of STAT1 in *IFNAR1* ^−*/*−^ cells impacted upon the ability of IFNγ to protect them against infection by the cytopathic virus murine encephalomyocarditis virus (EMCV). *Wild-type*, *IFNAR1* ^−*/*−^, *IFNAR1* ^−*/*−^ *MSCV*, and *IFNAR1* ^−/−^ *HA-STAT1* MEFs were infected with a dose of virus sufficient to induce 100% lysis of *wild-type* MEFs in the presence or absence of various doses of IFNγ, and the cytopathic effects were determined by assessing cell viability after 24 h. As was shown previously [5], the ability of IFNγ to protect cells from EMCV-mediated lysis was significantly reduced in *IFNAR1* ^−*/*−^ MEFs when compared to *wild-type* MEFs at most doses of IFNγ and the concentration of IFNγ (500 IU/ml) required to provide 80% protection from the virus for *IFNAR1* ^−*/*−^ cells was much greater than that required to provide a similar level of protection for *wild-type* cells (10 IU/ml). The response of *IFNAR1* ^−*/*−^ MEFs transduced with empty vector to IFNγ was not significantly different from the untransduced *IFNAR1* ^−*/*−^ MEFs at any dose of IFNγ and the concentration of IFNγ required to provide 80% protection (450 IU/ml) was of a similar order of magnitude (Figure 7). In contrast, protection from virus-induced lysis was significantly enhanced in *IFNAR1* ^−*/*−^ *HA-STAT1* MEFs at most doses of IFNγ. These data provide direct evidence that the attenuated protective anti-viral responses to IFNγ observed in *IFNAR1* ^−*/*−^ cells is a consequence of reduced STAT1 expression.

## Discussion

**Figure 8:**
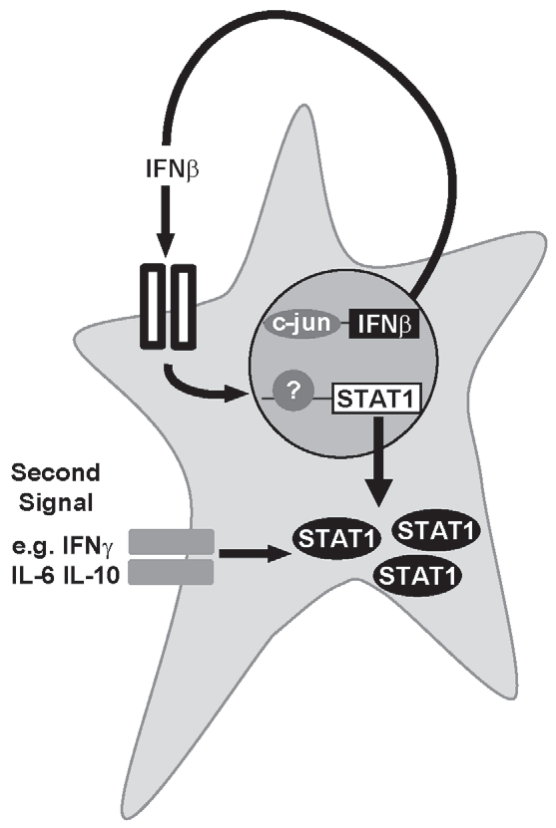
STAT1 levels are regulated by autocrine/paracrine stimulation by type I IFN. c-Jun regulates secretion of small amounts of type I IFN, which then stimulate adjacent cells and induce the expression of STAT1. The level of STAT1 that is expressed by a cell dictates how it responds to other stimuli, such as IFNγ, and other cytokines.

Herein we demonstrate that c-Jun is essential for the constitutive production of small quantities of IFNβ that initiates autocrine or paracrine feedback loops required to maintain the expression of STAT1 (Figure 8). This system was disrupted either by c-Jun deficiency, which prevents production of IFNβ, or by IFNAR deficiency, which affects the ability of cells to respond to the autocrine stimulus. Consistent with our data, others found that cells lacking IFNβ also express much lower levels of STAT1 [22] and virus-mediated induction of STAT1 is dependent on type I IFN signaling [11]. As IFNγ signaling is attenuated when the autocrine stimulus is blocked (Figure 8) but restored by adding back STAT1, it appears the level of STAT1 expressed by the cell determines the response of the cell to other cytokines. These results suggested the ability of IFNγ to induce a protective anti-viral state was due to the type I IFN-mediated maintenance of STAT1 expression rather than the recruitment of IFNAR1 into the IFNγR complex as has been previously proposed [5]. These findings define a novel mechanism through which STAT1-mediated signals can be regulated and highlight the importance of crosstalk between type I and II IFNs for anti-viral immunity.

It has been known for some time that, as well as being produced in large quantities following viral infections, cells can secrete low levels of type I IFN constitutively [1],[2],[27]. Virus-induced activation of the IFNβ enhanceasome is one of the best-characterized transcriptional modules [18],[23]. Viral activation of the IFNβ promoter involves the binding of NFκB, IRF3, and ATF2/c-Jun complexes to a series of DNA elements termed PRD I-IV [23]. In this setting, c-Jun binds to PRD IV of the promoter and facilitates co-operative binding of the other factors. Removing PRD IV from the promoter, or even reversing its orientation, has a major impact on the transcriptional activity of the promoter [23], suggesting the role of c-Jun is critical in the context of viral infection. In contrast, little is known of the molecular mechanisms of constitutive type I IFN production. Our study indicates that PRD IV of the IFNβ promoter is occupied by c-Jun even in “resting” cultured cells (Figure 4B). This requirement for c-Jun explains why we found that constitutive IFNβ production and hence the expression of STAT1 was attenuated in *c-Jun* ^−*/*−^ cells. In addition to regulating basal expression of IFNβ, we have recently demonstrated that c-Jun is activated following IFNγ treatment and may also play a direct role in regulating the expression of a subset of IFNγ-responsive genes (ISGs) [17]. Indeed we identified ISGs that were dependent on c-Jun for induction by IFNγ, others that required STAT1, and others that required both c-Jun and STAT1 for increased expression following treatment with IFNγ [17]. These results, coupled with the functional data provided herein, highlight the complex molecular interplay between c-Jun and canonical mediators of type I and II IFN signaling such as STAT1 in regulating a comprehensive response to IFN treatment.

Takaoka and colleagues previously demonstrated the importance of IFNβ in the production of an IFNγ-mediated anti-viral response [5]. In that paper the authors showed that *IFNβ* ^−/−^ MEFs were defective in mounting an IFNγ-induced antiviral response. These data mirror what we have demonstrated herein where we show that *IFNAR1* ^−/−^ MEFs show a similar defect in mounting an IFNγ-induced antiviral response. However, we showed that restoring STAT1 expression in *IFNAR1* ^−/−^ cells significantly rescued the ability of IFNγ to protect cells against EMCV, suggesting that regulating the levels of STAT1 expression through the autocrine loop may play an important role in responses to this challenge. The ability of type I and II IFNs to co-operate, for example, in treatment of melanoma tissue [28] or priming of macrophage cytotoxicity [29] has long been recognized. Interestingly, at a cellular level, *IFNAR1* ^−*/*−^ cells were known to have an anomalously poor response to IFNγ with respect to induction of GAS DNA binding, induction of gene expression, and protection against the cytopathic effects of EMCV [4],[5],[25]. IFNγ function is not entirely compromised in *IFNAR1* ^−*/*−^ animals because *IFNGR1* ^−*/*−^ mice have distinct phenotypic differences from *IFNAR1* ^−*/*−^ mice [30]. Inhibiting autocrine priming by type I IFN does not only affect signaling by IFNγ. Therefore its is not surprising that IL-6 signaling [31] and CSF-1 signaling are affected by inhibiting priming by type I IFN [2] and that signals induced by IL-10 can be affected by priming with IFNs [32].

It was proposed that the ligand-bound IFNAR1 chain acts as a component of the IFNGR and promotes recruitment of STAT1 to the IFNGR because IFN receptors are clustered within caveolar membrane fractions to facilitate their association [5]. Such a hypothesis is inconsistent with mapping of the docking site of STAT1 to the IFNAR2 chain of the type I IFN-R rather than the IFNAR1 as specified by the shared receptor model [33]. We demonstrated herein that *IFNAR1* ^−*/*−^ cells express lower basal levels of STAT1 relative to *wild-type* controls (Figure 5), and as STAT1 is a critical mediator of IFN signaling, this is an alternative reason why these cells may lack sensitivity to IFNγ. Our model not only explains the inability of IFNγ to prime *IFNAR1* ^−*/*−^ cells for an anti-viral response and the rescue of IFNγ function in *IFNAR1* ^−*/*−^ cells by STAT1α expression but also the attenuated responses to other cytokines, such as IL-6 and CSF-1, observed in *IFNAR1* ^−/−^ cells [2],[31] and predicts they may also be rescued by expression of STAT1. As IFNγ function was not entirely recovered following re-expression of STAT1 in *IFNAR* ^−*/*−^ cells, we cannot exclude that the shared receptor mechanism makes a contribution, but there are other reasons why reconstitution of STAT1 may not have fully rescued IFNγ function. These include the absence of other as yet unidentified signal transducing proteins from cells of this genotype.

The level of STAT1 expression in cells can have functional consequences with respect to immune responses. In response to viral infection, Ag-specific CD8^+^ T cells express peak levels of STAT1 for a shorter period of time than CD4^+^ cells [34]. This decreased sensitivity to IFN-induced growth inhibition allows expansion of Ag-specific CD8^+^ cells while the proliferation of cells with higher STAT1 is inhibited [34]. The relative amounts of different STATs can also affect the biological responses to cytokines. For example STAT1:3 and STAT1:4 ratios have been shown to alter cellular responses, and thus regulating the levels of these transcription factors will affect the outcome of immune responses [11],[35].

Our previous studies revealed that loss of IFN signaling abrogated the immune-mediated neo-natal lethality of SOCS1^−/−^ deficiency [36], and more recently we discovered that deleting *IFNAR1* also rescued this pathology [37] to a level equivalent to *SOCS1* ^−*/*−^ *IFN^+/^* ^−^. Although SOCS1 directly regulated type I IFN signaling, another reason why *IFNAR1* deficiency can protect SOCS1^−/−^ animals may be the similarities and crosstalk between type I and II IFN signaling pathways. These data highlight the patho-physiological importance and mechanism of crosstalk between type I and II IFN that are important considerations in understanding the contributions of individual cytokines to host defense and in their therapeutic targeting.

## Materials and Methods

### Cells and Reagents

*c-Jun* ^−*/*−^, *IFNAR1* ^−*/*−^ [38], *IFNAR2* ^−*/*−^ [39], and *wild-type* matched MEFs were derived from embryos and either used as early passage primary MEFs or immortalized by the “3T3” method. *IFNAR1* ^−*/*−^ MEFs were transduced with Murine stem cell leukemia virus supernatants encoding GFP alone or cDNA encoding HA-tagged STAT1 (generous gift from Thomas Decker). Supernatants were produced by transient transfection of PhoenixE cells with MSCV vector by calcium phosphate precipitation using standard methods.

*c-Jun* ^−*/*−^ MEFs were transduced with lentiviral supernatants encoding miR sequences targeting IFNβ (Open Biosystems, Huntsville, AL, USA; product numbers: RMM4431-98755134, RHS4346). Supernatants were produced by transient transfection of 293T cells with pGIPZ vector using Lentiphos HT kit (Clontech, Mountain View, CA, USA) according to the manufacturer's instructions.

Cells were cultured in DMEM supplemented with 5% foetal bovine serum (JRH Biosciences, Lenexa, KS, USA) and 2 mM L-Glutamine (JRH Biosciences, Lenexa, KS, USA). All tissue culture reagents were certified sterile and free of Mycoplasma and pyrogens. Antibodies for the following targets were used: STAT1 (BD Biosciences Franklin Lakes, NJ, USA), STAT3 (Santa Cruz Biotech, Santa Cruz, CA, USA), HA (Cell Signaling Technology, Beverly, MA, USA), c-Jun (Santa Cruz Biotech, Santa Cruz, CA, USA), α-tubulin (Sigma Chemical Co., St. Louis, MO, USA), and hsp70 (Clone N6 was a kind gift of Dr. Robin Anderson; Peter MacCallum Cancer Centre, Melbourne, Australia). Neutralizing anti type I IFN [20] and anti-IFNAR1 were described previously [40]. HRP-conjugated secondary antibodies were purchased from Dako (Glostrup, Denmark).

### Production of Conditioned Media

3×10^6^ MEFs were cultured in 175 cm^2^ tissue culture flasks in 20 mL media for 3 d. Supernatant was collected, cell debris removed by centrifugation (670 g, 4 min), sterilized using a 0.22 µM filter, and stored at 4°C.

### SDS-PAGE and Western Blotting

Western blotting was performed as previously described [17]. Briefly, cells were washed, resuspended in whole cell lysis buffer (50 mM Tris-HCl pH 8, 0.1% Triton X-100, 150 mM NaCl, 0.1 mM EDTA, 0.1 mM EGTA, 10% glycerol, 1 µg/mL aprotinin, 0.5 µg/mL leupeptin, and 0.2 mM PMSF), and after (4°C 10 min) lysates were cleared by centrifugation. Proteins were separated by SDS-PAGE, transferred to immobilon P membranes (Millipore), and probed with specific antibodies. Secondary antibodies were conjugated to horseradish peroxidase and images were visualized by chemiluminescence (ECL, GE Healthcare, Bucks, UK).

### Nuclear Extraction and EMSA

Nuclear extractions and EMSAs were performed as previously described [17]. Briefly, cells were resuspended in hypotonic lysis buffer (10 mM HEPES, 1.5 mM MgCl_2_, 10 mM KCl, and protease inhibitors) (4°C, 5 min), NP-40 was added to a final concentration of 0.25%, and the nuclei isolated by centrifugation (2,000 g, 10 min). Nuclei were resuspended in hypertonic lysis buffer (5 mM HEPES pH 8, 1.5 mM MgCl_2_, 0.2 mM EDTA, 0.5 M NaCl, 25% glycerol, and protease inhibitors) (4°C, 1 h). For binding reactions 5–10 µg of nuclear lysate was incubated (4°C, 30 min) with 5×10^4^ cpm T4 PNK-^32^P-labeled oligonucleotides in binding buffer (20 mM Tris/HCl pH 8, 6 mM KCl, 2 mM MgCl_2_, 12% Glycerol, 5 µM DTT, 2.5 µg polydI.dC.polydI.dC, and 0.05% NP-40). Complexes were separated by 5% native PAGE, and gels were dried and visualized by autoradiography on X-ray film (Kodak). The sequence of the GAS oligonucleotide 5′-TAGGGATTTACGGGAAATTGATGAAGCTGATC-3′ was derived from the FcγRI promoter; the AP-1 oligonucleotides were described previously [17].

### Quantitative Real Time PCR

RNA was extracted using Trizol (Invitrogen, Carlsbad, CA, USA) according to the manufacturer's instructions. cDNA was synthesized from 2 µg RNA using superscript III (Invitrogen, Carlsbad, CA, USA) as per the manufacturer's instructions. The abundance of specific genes in the samples was quantitated using the SYBR Green dye detection method (Applied Biosystems, Foster City, CA, USA). Primers to murine *GBP-1* (5′-TGTGGTTGCTGGATGAGCAGAGTA-3′; 5′-AAGGAAACACAGTAGGCTGGAGCA-3′), *SOCS3* (5′-CCTTCAGCTCCAAAAGCGAG-3′; 5′-GCTCTCCTGCAGCTTGCG-3′), and IFNβ (5′-AGCTCCAAGAAAGGACGAACAT-3′; 5′-GCCCTGTAGGTGAGGTTGATCT-3′) were designed using Primer Express 2 software (Applied Biosystems, Foster City, CA, USA). Primers to murine STAT1 gene (5′-CGCGCATGCAAGTGGCATATAACT-3′; 5′-AAGCTCGAACCACTGTGACATCCT-3′) were designed using PrimerQuest software (Integrated DNA Technologies). Primers to ribosomal *L32* (5′-TTCCTGGTCCACAACGTCAAG-3′; 5′-TGTGAGCGATCTCGGCAC-3′) were as previously described [17]. Threshold cycle numbers (Ct) were measured in the exponential phase for all samples. Relative abundance of sample genes was calculated using the ΔΔCt method relative to the L32 control gene [17]. mRNA abundance was normalized to the untreated samples of each genotype.

### ChIP

ChIP assays were performed as described previously [41] using 5 µg of anti c-Jun or rabbit IgG control antibodies. The abundance of specific sequences in ChIP samples was quantitated using the SYBR Green dye detection method (Applied Biosystems, Warrington, UK). Primers used for PCR reactions were mIFN PRDIV (5′-ATTCCTCTGAGGCAGAAAGGACCA; 5′-GCAAGATGAGGCAAAGGCTGTCAA) and were designed using Primer Express 2 software. Threshold cycle values (Ct) were measured in the exponential phase, and promoter occupancy was calculated using the formula 2^(Ct Ig − Ct c-Jun)^.

### Statistical Analysis

Statistical significance was tested using one-way ANOVA testing with OriginLab 7.5 software (Northampton, MA, USA) or Prism Software Graphpad (La Jolla, CA, USA).

### Viral Protection Assays

10^3^ cells of each genotype were plated in duplicate wells in a 96 well plate and allowed to adhere. Media was replaced with fresh media containing murine EMCV (M.O.I of 0.1) and various concentrations of IFNγ (0–1,000 IU/mL) and cultured for 16 h. As controls, cells were cultured in fresh media alone (100% survival) or with EMCV alone (0% survival). Cells were washed in PBS, formalin fixed (10 min at RT), washed (twice with PBS), and stained in 0.5% Crystal Violet/20% methanol. Stained cells were extensively washed, crystal violet was solubilized in 10% acetic acid, and OD_550_ nm was recorded. Viability was calculated by comparison against a standard curve.

## Supporting Information

Figure S1 **STAT1 expression is decreased in c-Jun knockout cells**. SDS-PAGE and Western blotting with antibodies against STAT1 was performed using whole cell extracts from an independently derived set of *wild-type* or *c-Jun* ^−*/*−^ MEFs. As a control, the expression of α-tubulin was also tested by Western blot.
(0.35 MB TIF)

Figure S2 **IFNβ regulates the expression of STAT1.** (A) *c-Jun* ^−*/*−^ MEFs were treated with various doses of IFNβ for 24 h, RNA was isolated, and expression of STAT1 was assessed by qRT-PCR. STAT1 expression in untreated *wild-type* MEFs was also assessed as a control. Histograms represent mean and error bars the SEM of three independent experiments and are expressed relative to the levels detected in *c-Jun* ^−*/*−^ cells (arbitrarily set as one). (B) c*-Jun* ^−*/*−^ MEFs were treated in the presence or absence of 10 IU IFNβ for 24 h, cell lysates were prepared, and expression of STAT1 was assessed by Western blot. (C) *Wild-type* MEFs were treated in the presence or absence of various doses of IFNβ for 24 h, RNA was isolated, and expression of STAT1 was assessed by qRT-PCR. Histograms represent mean and error bars the SEM of three independent experiments (arbitrarily set as one).
(0.81 MB TIF)

Figure S3 **Expression of STAT1, 2, and 3 in IFNAR1 knockout cells.** (A) The expression of STAT2 mRNA (top panel) and STAT3 protein (bottom panel) was determined in *wild-type* and *IFNAR1* ^−*/*−^ MEFs by QRT-PCR and Western blotting, respectively. (B) *Wild-type*, *IFNAR1* ^−*/*−^, or *c-Jun* ^−*/*−^ MEFs were cultured in fresh media (white bars) or conditioned media from *wild-type* MEFs (black bars) for 16 h. RNA was extracted, cDNA synthesized, and STAT1 mRNA expression assessed by qRT-PCR. *STAT1* mRNA expression was normalized to that of untreated IFNAR1^−/−^ MEFs cultured in fresh media. Data are representative of three similar experiments.
(0.65 MB TIF)

Figure S4 **Reconstitution of STAT1 in IFNAR1 knockout cells restores IFNγ-mediated upregulation of IFN response genes.** *Wild-type* MEFs, IFNAR1^−/−^ MEFs, and *IFNAR1* ^−/−^ MEFs transduced with empty vector (IFNAR1^−/−^ MSCV) or IFNAR1^−/−^ MEFs transduced with HA-tagged STAT1α (IFNAR1^−/−^ STAT1) were treated with 100 IU/mL IFNγ for 0, 1, or 6 h. RNA was extracted, cDNA synthesized, and qRT-PCR performed with primers specific for *caspase 4 (CASP 4)*, *CISH*, *CXCL11*, and *MYD88*. mRNA levels are expressed relative to those of *wild-type* C57/BL6 (B6) splenocytes. Histograms represent the mean and error bars the standard error of four independent experiments (* *p*<0.05 for samples that were significantly induced).
(1.56 MB TIF)

## Abbreviations

ChIP: chromatin immunoprecipitation
EMCV: encephalomyocarditis virus
EMSA: elecrophoretic mobility shift assay
GAS: gamma activated sequence
IFN: interferon
IFNAR1: IFNα/β receptor1
IFNGR: IFNγ receptor
IL: interleukin
ISG: IFN-stimulated gene
JAK: Janus family kinase
MEFs: murine embryonic fibroblasts
PIAS: Protein Inhibitor of Activated STAT
RNAi: RNA interference
