# Supplementary figures and images for: Functional Crosstalk between Type I and II Interferon through the Regulated Expression of STAT1
Source: PLoS Biol. 2010 Apr 27;8(4):e1000361. doi: 10.1371/journal.pbio.1000361 (PMC2860501; doi:10.1371/journal.pbio.1000361)

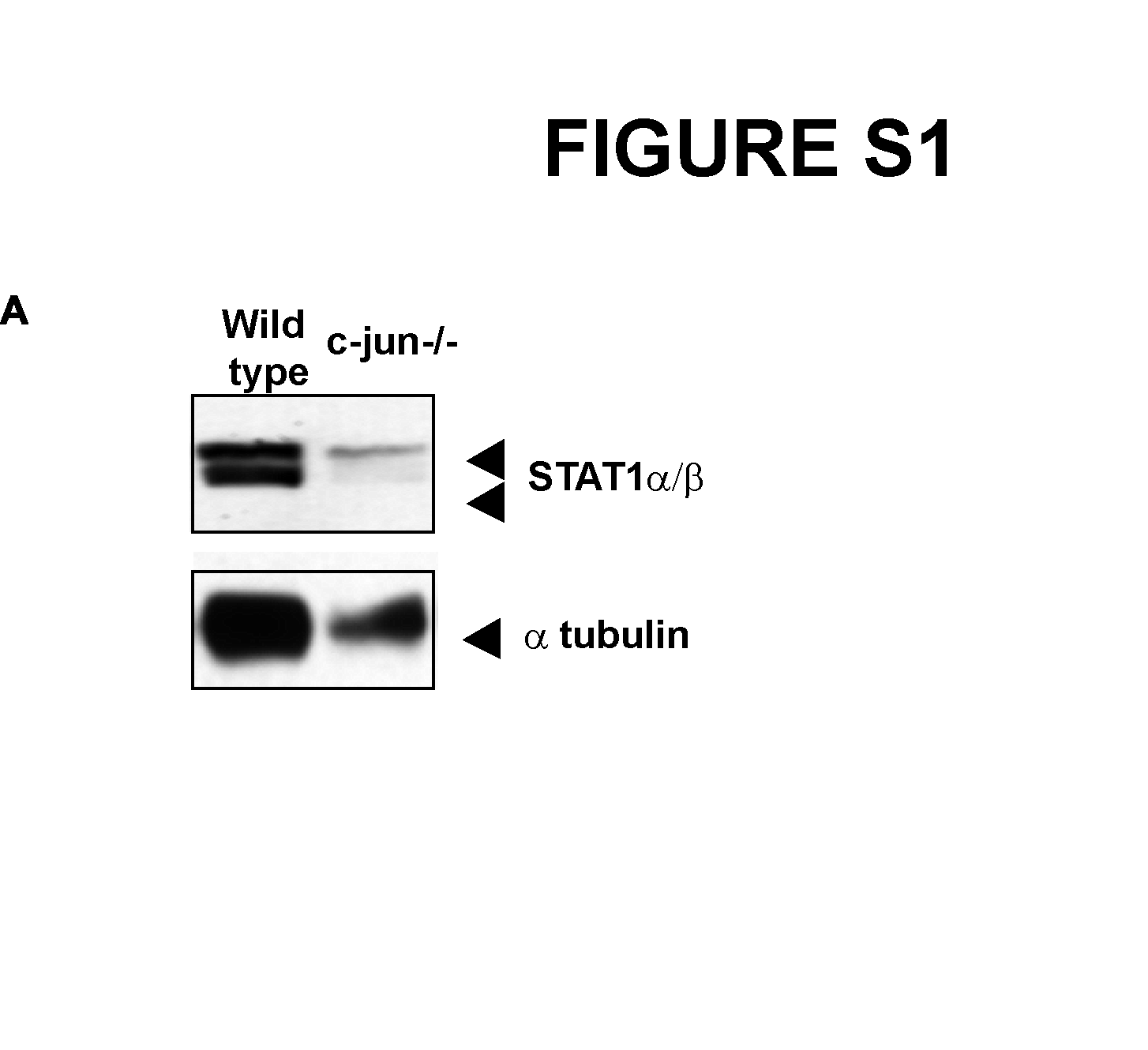

Supplement: Figure S1 — STAT1 expression is decreased in c-Jun knockout cells. SDS-PAGE and Western blotting with antibodies against STAT1 was performed using whole cell extracts from an independently derived set of wild-type or c-Jun −/− MEFs. As a control, the expression of α-tubulin was also tested by Western blot. (0.35 MB TIF) [file pbio.1000361.s001.tif]

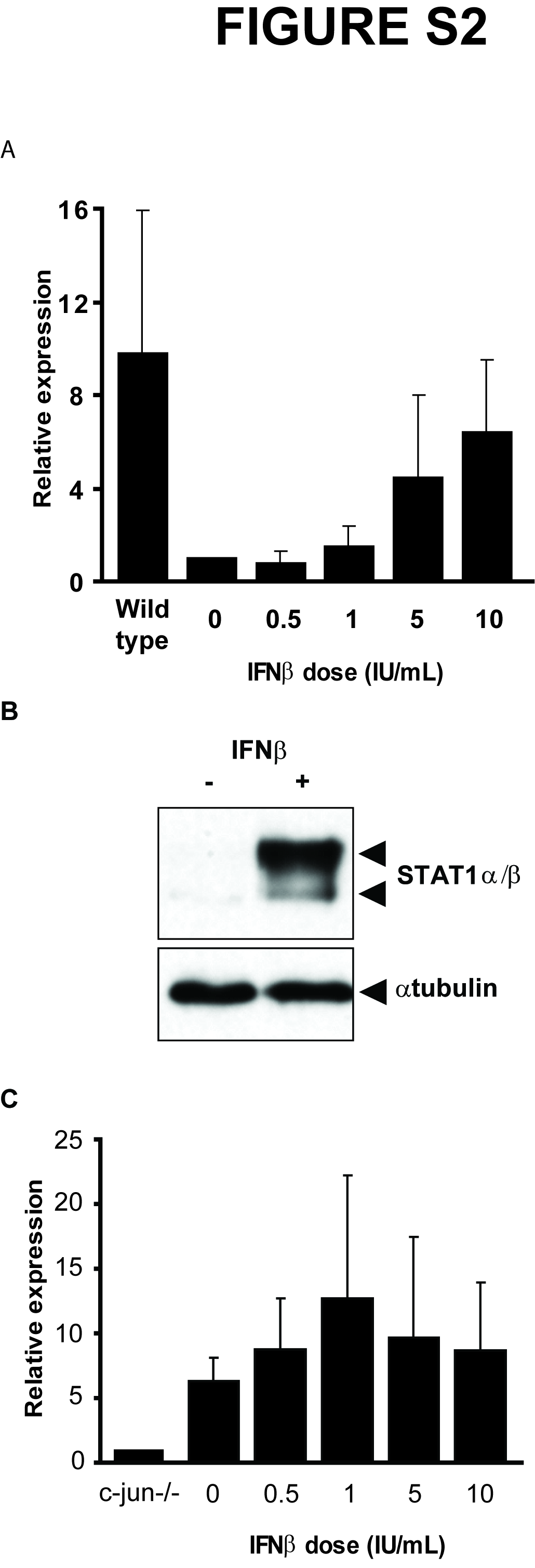

Supplement: Figure S2 — IFNβ regulates the expression of STAT1. (A) c-Jun −/− MEFs were treated with various doses of IFNβ for 24 h, RNA was isolated, and expression of STAT1 was assessed by qRT-PCR. STAT1 expression in untreated wild-type MEFs was also assessed as a control. Histograms represent mean and error bars the SEM of three independent experiments and are expressed relative to the levels detected in c-Jun −/− cells (arbitrarily set as one). (B) c-Jun −/− MEFs were treated in the presence or absence of 10 IU IFNβ for 24 h, cell lysates were prepared, and expression of STAT1 was assessed by Western blot. (C) Wild-type MEFs were treated in the presence or absence of various doses of IFNβ for 24 h, RNA was isolated, and expression of STAT1 was assessed by qRT-PCR. Histograms represent mean and error bars the SEM of three independent experiments (arbitrarily set as one). (0.81 MB TIF) [file pbio.1000361.s002.tif]

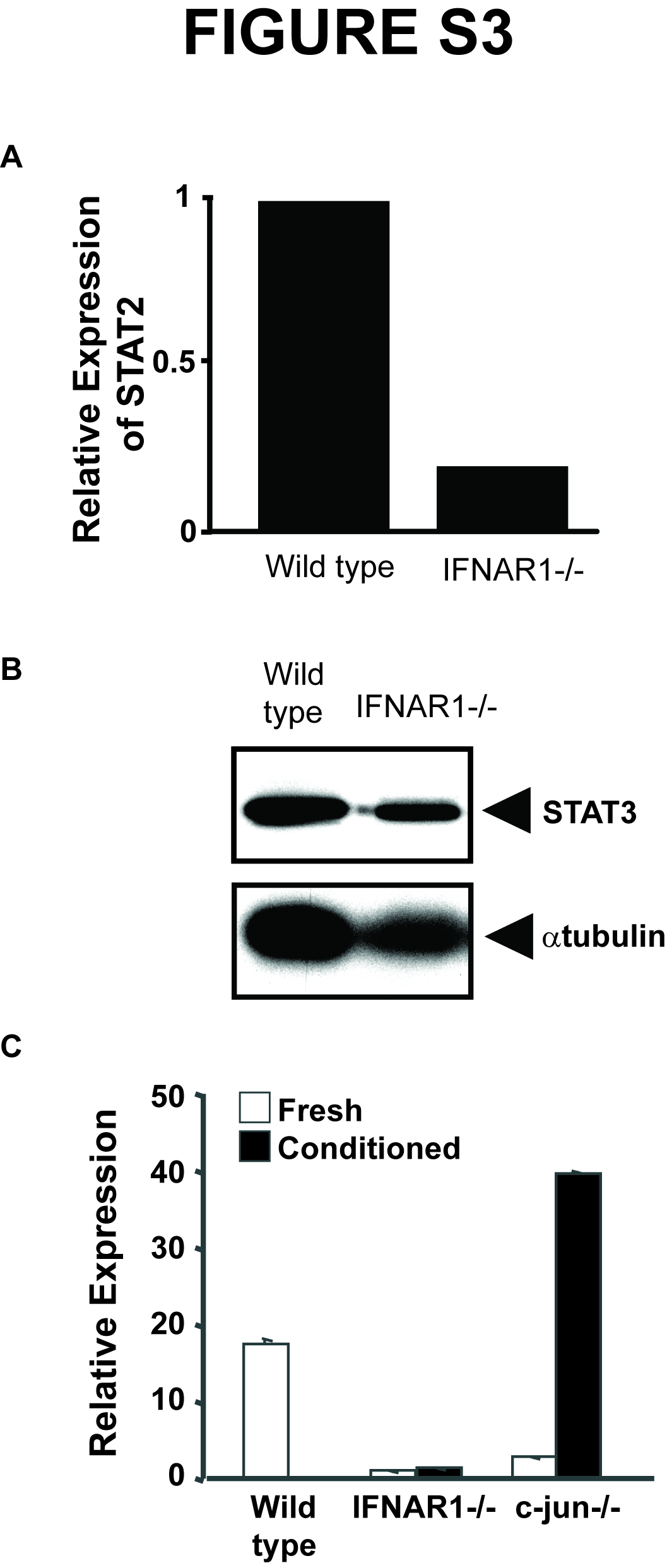

Supplement: Figure S3 — Expression of STAT1, 2, and 3 in IFNAR1 knockout cells. (A) The expression of STAT2 mRNA (top panel) and STAT3 protein (bottom panel) was determined in wild-type and IFNAR1 −/− MEFs by QRT-PCR and Western blotting, respectively. (B) Wild-type, IFNAR1 −/−, or c-Jun −/− MEFs were cultured in fresh media (white bars) or conditioned media from wild-type MEFs (black bars) for 16 h. RNA was extracted, cDNA synthesized, and STAT1 mRNA expression assessed by qRT-PCR. STAT1 mRNA expression was normalized to that of untreated IFNAR1−/− MEFs cultured in fresh media. Data are representative of three similar experiments. (0.65 MB TIF) [file pbio.1000361.s003.tif]

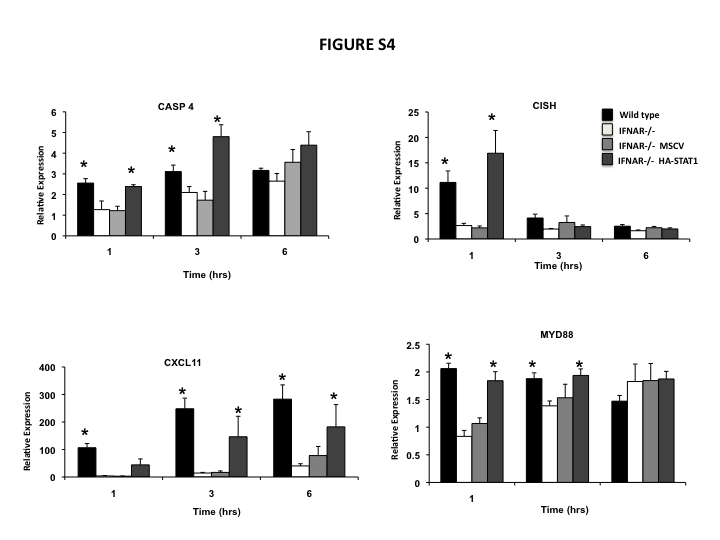

Supplement: Figure S4 — Reconstitution of STAT1 in IFNAR1 knockout cells restores IFNγ-mediated upregulation of IFN response genes. Wild-type MEFs, IFNAR1−/− MEFs, and IFNAR1 −/− MEFs transduced with empty vector (IFNAR1−/− MSCV) or IFNAR1−/− MEFs transduced with HA-tagged STAT1α (IFNAR1−/− STAT1) were treated with 100 IU/mL IFNγ for 0, 1, or 6 h. RNA was extracted, cDNA synthesized, and qRT-PCR performed with primers specific for caspase 4 (CASP 4), CISH, CXCL11, and MYD88. mRNA levels are expressed relative to those of wild-type C57/BL6 (B6) splenocytes. Histograms represent the mean and error bars the standard error of four independent experiments (* p<0.05 for samples that were significantly induced). (1.56 MB TIF) [file pbio.1000361.s004.tif]
